# Retaining well-fixed cementless stem in the treatment of infected hip arthroplasty: Good results in 19 patients followed for mean 4 years

**DOI:** 10.3109/17453674.2013.795830

**Published:** 2013-05-31

**Authors:** Young-Kyun Lee, Kee Haeng Lee, Jae-Hwi Nho, Yong-Chan Ha, Kyung-Hoi Koo

**Affiliations:** ^1^Department of Orthopaedic Surgery, Seoul National University Bundang Hospital, Seongnam-si; ^2^Department of Orthopaedic Surgery, Bucheon St. Mary’s Hospital, The Catholic University of Korea, Bucheon-si; ^3^Department of Orthopaedic Surgery, Soonchunhyang University Hospital Cheonan, Cheonan-si; ^4^Department of Orthopaedic Surgery, Chung-Ang University College of Medicine, Seoul, South Korea.

## Abstract

**Background and purpose:**

Two-stage reconstruction, reimplantation after removal of an infected prosthesis, has been considered to be the gold standard for treatment of infected hip arthroplasty. However, during the removal of a well-fixed femoral stem, the proximal femur can be damaged and a sequestrum can be formed, which might lead to chronic osteomyelitis and difficulty in reimplantation. We wanted to determine whether infection after hip arthroplasty can be treated without removal of a well-fixed stem.

**Methods:**

We treated 19 patients who had an infection after hip replacement, but a well-fixed cementless stem, with 2-stage reconstruction. At the first stage, we removed the acetabular cup, the liner and the head, but not the stem. We then implanted a cup of cement spacer. After control of infection, we reimplanted the acetabular component and head.

**Results:**

2 patients did not undergo second-stage reconstruction because they were satisfied with the pain relief and the activity that they had with the cement-spacer implantation. The remaining 17 patients underwent the second-stage of the reconstruction using cementless arthroplasty. At a mean follow-up time of 4 (2–8) years, 15 of the patients had no recurrence of infection, with satisfactory clinical and radiographic outcome.

**Interpretation:**

This second-stage reconstruction after retention of the stem could be an alternative treatment option for periprosthetic infection with a well-fixed stem.

Infection following total hip arthroplasty is a disastrous complication. Since its introduction in 1993, 2-stage reimplantation using an interval spacer of antibiotic-impregnated bone cement has been considered the gold standard for treatment of infected hip arthroplasties (Lieberman et al. 1994, Tsukayama et al. 1996, Masri et al. 2007, Beswick et al. 2012).

It is generally believed that all components should be removed to eradicate the infection. However, an extended trochanteric osteotomy or cortical window is often required to remove a well-fixed femoral stem. This necessitates an extensive soft tissue dissection that devascularizes the proximal femur, which may lead to formation of sequestrum, causing recurrence of infection. If a circumferential bone ingrowth of a cementless stem acts as a barrier to intrusion of infected joint fluid and microorganisms, the infection could be treated without the removal of a well-fixed stem, and the risk of recurrent infection and implant failure would be lower.

There have been no reports on the results of stem-retaining 2-staged revision. We hypothesized that infection after hip arthroplasty could be treated without removal of a well-fixed cementless stem. We assessed the outcome of such a procedure in 19 patients.

## Patients and methods

From January 2005 to April 2010, 38 patients were operated with 2-stage revision to treat an infection after hip arthroplasty. A diagnosis of infection was made when there was drainage of pus, positive culture of aspirated ﬂuid and/or tissue, or histological evidence of infection (Lonner et al. 1996, Estes et al. 2010).

Our basic principle was 2-stage revision: removal of implants, debridement, and insertion of antibiotic-impregnated cement spacers in the first stage and implantation of new prostheses in the second stage (Koo et al. 2001). In the first stage, we removed acetabular components, heads, cemented stems, and loose cementless stems without exception.

In cases of well-fixed cementless stems with radiographic evidence of bone ingrowth in the proximal one third or less and radiolucent lines around the distal portion of the stem (Engh et al. 1990, Kim and Kim 1993), we tried to remove the stem by making a cleavage between the stem and the proximal femur using thin osteotomes and gauges, and then pulling out the stem using a stem extractor. If this procedure did not work, we did not remove the stem. In cases where there was evidence of bone ingrowth along the entire length of the stem on the preoperative radiographs, we did not attempt stem removal because an extended osteotomy would be required to remove the stem with the risk of femoral fracture when the stem was being pulled out.

According to the above principle, 19 patients in 3 hospitals (7 men and 12 women, 19 hips) were treated without removal of their cementless stem; they were the subjects of this study. The stem was retained after a failed attempt at stem removal in 9 patients, while stem removal was not attempted in 10 patients.

Mean age at operation was 60 (31–85) years. 9 patients had undergone primary total hip arthroplasty, 7 patients bipolar hemiarthroplasty, and 3 patients revision hip arthroplasty. The mean time interval between the previous hip arthroplasty and the onset of symptoms of infection was 4 (0.5–12) years. Type of infection was defined as follows. An early postoperative infection was an infection that developed less than 1 month after the operation. A late chronic infection was one that developed 1 month or more after the index operation and which had an insidious clinical course. An acute hematogenous infection was associated with a documented or suspected antecedent bacteremia and was characterized by an acute onset of symptoms in the affected joint with the prosthesis (Tsukayama et al. 1996, McPherson et al. 2002). The types of infection were late chronic infection in 10 patients and acute hematogenous infection in 9. 6 patients had been treated previously with antibiotics following incision and debridement elsewhere (Table). The mean preoperative Harris hip score was 60 (33–86) points.

**Table:** Characteristics of patients who underwent two-stage reconstruction

A	B	C	D	E	F	G	H	I	J
1	M/63	primary THA	AML stem	No	96	late chronic	6	*S. aureus*	69
2	F/58	primary THA	AML stem	No	146	late chronic	13	Culture neg.	24
3	F/31	stem revision	Corail stem	I & D	7	acute hematogenous	4	*S. aureus*	31
4	M/39	primary THA	CLS stem	I & D	85	late chronic	8	CNS	39
5	F/64	primary THA	Euroform stem	No	62	late chronic	24	*S. aureus*	24
6	F/69	primary THA	Corail stem	No	6	acute hematogenous		Streptococci	10
7	M/56	HA	CLS stem	I & D	216	late chronic	9	*S. aureus*	71
8	F/36	primary THA	AML stem	I & D	39	late chronic	3	Culture neg.	100
9	F/56	total revision	Bicontact stem	I & D	13	late chronic	15	Culture neg.	88
10	F/37	primary THA	Gemini stem	No	173	late chronic	7	*S. aureus*	51
11	M/53	primary THA	Corail stem	No	6	acute hematogenous	4	CNS	Recurred
12	M/59	primary THA	Anatomical 6° stem	No	144	acute hematogenous	7	Streptococci	Recurred
13	F/74	HA	Summit stem	No	9	late chronic	3	*S. aureus*	26
14	M/85	HA	Summit stem	No	9	acute hematogenous	2	Enterococci	26
15	F/74	HA	Summit stem	No	9	acute hematogenous	1	CNS	43
16	M/51	cup revision	Bicontact stem	No	19	acute hematogenous	4	CNS	26
17	F/84	HA	Bencox II stem	No	38	acute hematogenous	2	Culture neg	24
18	F/79	HA	Soma stem	No	36	acute hematogenous		*S. aureus*	25
19	F/77	HA	Wagner stem	I & D	72	late chronic	2	CNS	40

THA: total hip arthroplasty; HA: hemiarthroplasty; I & D: incision and debridement; CNS: coagulase-negative *Staphylococcus.* A Patient no. B Sex/age C Type of index arthroplasty D Previous stem E Previous treatment F Interval between index arthroplasty and first-stage revision (months) G Type of Infection H Interval between procedures (months) I Etiological organism F Duration of follow-up (months)

In 10 patients, the infecting organisms were identified from the drainage or aspirated fluid before the first stage of revision. Of the remaining 9 patients, the infecting organism was identified during the first-stage procedure in 5 (Table). The organisms identified were sensitive to at least 1 of the 3 antibiotics vancomycin, tobramycin, and cefotaxime, which were present in the impregnated cement spacer in the present study.

### Operative and postoperative procedures

The ﬁrst procedure consisted of removal of the acetabular cup, liner, head, and other foreign materials such as wire; debridement of dead tissue; and insertion of a spacer. All operations were done using a posterolateral approach. During the procedure, several samples (at least 3) were sent for culture and sensitivity tests, and histological evaluation. Osteolytic lesions, if present, were curetted. The removed head was washed and immediately sterilized by autoclaving for 15 min and betadine-soaking.

**Figure 1.**
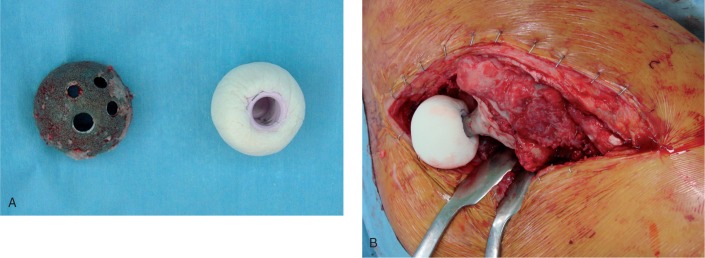
A. The cement spacer was molded in the shape of a hemiarthroplasty prosthesis over the sterilized head, according to the size of the retrieved acetabular cup. B. The molded cement spacer was attached to the retained femoral stem.

For the hand-molded cement spacer, we used a so-called cocktail regimen of 3 antibiotics: vancomycin, tobramycin and cefotaxime (Lieberman et al. 1994, Koo et al. 2001, McPherson et al. 2002, Leung et al. 2011). 6 g of antibiotics (3 × 2 g) was pulverized and mixed thoroughly with 2 batches (80 g in total) of polymethylmethacrylate polymer (Simplex P; Stryker, Kalamazoo, Michigan). Then, the contents of 2 ampules of liquid monomer (40 mL in total) were added to the mixture. During the doughy stage of polymerization, the mixture was hand-molded into a hemispheric shape around the sterilized head, according to the diameter of the acetabular cup (Figure 1A). The head in the constructed cement spacer was fitted onto the stem trunion. The bare area of the stem surface was covered with about 4 mm of antibiotic-impregnated cement (Figure 1B). The cement-spacer cup was then reduced into the acetabulum. In 5 hips, small cement beads were implanted in dead spaces around the acetabulum and the proximal femur.

A closed suction drainage was inserted, and it was removed 2–5 days after the operation when the amount of daily drainage was less than 50 mL. After the removal of the drain, the patients were permitted to walk, with weight bearing as tolerated.

Antibiotics for the postoperative treatment were selected on the basis of the sensitivities of the organism identiﬁed in the culture. Vancomycin was administered to 12 patients, cefazolin to 6, and ciproﬂoxacin to 1. These antibiotics were administered parenterally for 4–6 weeks after the first procedure. The interval between the 2 procedures was determined according to the improvement in clinical and laboratory findings of infection. Except for 1 patient (no. 15), who underwent the second-stage reconstruction 4 weeks after the first-stage procedure, the antibiotic-free interval was more than 1 month, and it was extended if the wound was slow to heal or ESR and/or CRP levels were slow to normalize (Table).

Of the 19 patients, 2 did not undergo second-stage reconstruction because their pain was tolerable and they were satisfied with the activity that they had obtained with the cement-spacer implantation.

In the remaining 17 patients, the second procedure was performed 1–24 months after the ﬁrst procedure. At the time of the second procedure, the spacer and antibiotic beads were removed and debridement of dead tissue was done. Several (at least 3) samples were sent for culture and sensitivity tests, and several synovial samples were examined for histology of frozen sections. During the second procedure, there was neither visible nor histological evidence of infection in any hips. No organisms were identiﬁed in culture of the samples obtained during the second-stage operation. 3 patients had bone-stock deﬁciency in the acetabulum, which was filled using morsellized bone grafts.

Cementless acetabular components and ceramic-on-ceramic articulation were used for the second-stage reconstruction. A secure press-fit was obtained in all hips.

The closed suction drain was removed 2 days after the second-stage operation, when draining volume was below 50 mL, and the patient was allowed to walk with 2 crutches for 1–3 months. Cefazolin was administered parenterally for 3 days after the second procedure.

Clinical and radiographic follow-up evaluations were performed at 2 and 6 weeks, at 3, 6, 9, and 12 months, and every 6 months thereafter. At the time of each follow-up, hematological studies were performed to check for recurrence of infection. Harris hip score was recorded (Harris 1969).

The fixation of the acetabular cup was assessed on AP and cross-table lateral radiographs using the method of Latimer and Lachiewicz (1996). The fixation of the femoral component was assessed using the methods of Engh et al. (1990) and Kim et al. (1993). The cup was considered loose when there was migration of > 2 mm or a change in the abduction of > 4°. The stem was considered definitely loose when there was a subsidence of > 3 mm, and possibly loose when there was a complete radiolucent line along the entire porous-coated surface on both the AP and lateral radiographs. Postoperative 6-week radiographs were used as baseline and the final radiographic analysis was performed at the last follow-up.

Failure of treatment was deﬁned as a recurrence of infection.

The design and protocol of this multicenter retrospective study were approved by the institutional review boards at each center, who waived informed consent.

## Results

After the first-stage procedure, acetabular erosion was seen around the cement spacer on radiographs. It was between 2 mm and 8 mm, and did not cause any problems in implantation of the acetabular cup in the second-stage procedure.

2 patients who did not undergo second-stage reconstruction (nos. 6 and 18) were followed for mean 1.5 (1–2) years after the first-stage operation. They had no recurrence of infection. Hip pain was minimal or mild, and they could walk with a cane and perform their daily activities.

**Figure 2.**
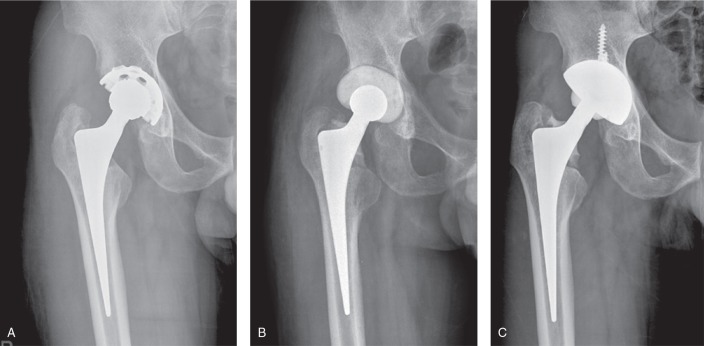
A. A 63-year-old man (patient no. 1) who had an infection after total hip arthroplasty, showing a bone-ingrown femoral stem. B. After the first-stage operation with an articulating cement spacer and retained femoral stem. C. 6 years after the second-stage reconstruction, with no evidence of implant loosening or osteolysis.

Of the 17 patients who underwent two-stage reconstruction, 15 had no recurrence of infection during the mean follow-up time of 4 (2–8) years after the second-stage operation (Figure 2). The mean Harris hip score improved to 89 (65–94) points at the latest follow-up. 1 patient had an osteolytic lesion around the lateral wall of the acetabulum, but that was not progressive. There was no loosening of acetabular or femoral components.

Infection recurred in 2 patients. 1 patient (no. 11), a 53-year old man who had diabetes mellitus and alcoholic liver cirrhosis, had a recurrence of infection 7 months after the second-stage reconstruction. He was treated with a repeat 2-stage operation without removal of the femoral stem. At the 18-month follow-up after the repeat surgery, the patient had no evidence of infection. He had no pain and could walk without any limp. The other patient (no. 12), a 61-year old man who had diabetes mellitus and hepatocellular carcinoma, had a recurrent infection 7 months after the first-stage operation. He underwent a Girdlestone arthroplasty and second-stage reimplantation but he died 3 months after the repeated reconstruction due to hepatic failure.

## Discussion

In the present study, the infection healed after initial treatment in 15 of 17 patients, which is similar to or higher than the rates reported in other series of 2-staged revision (Hsieh et al. 2005, Masri et al. 2007, Leung et al. 2011). Infection was eliminated in 1 more patient after repeated two-stage revision.

One-stage revision is an attractive treatment for infected hip arthroplasty, because it can reduce morbidity, it reduces costs, and it avoids the technical difficulties of staged revision surgery. However, it is effective only at an early stage of infection by a low-virulence organism and should be performed with caution in selected patients (Ure et al. 1998, Jackson and Schmalzried 2000, Yoo et al. 2009). A recent analysis, which compared 1-stage and 2-stage revisions to determine the treatment modality that would result in greater quality-adjusted life years using a decision analysis, favored the 1-stage revision (Wolf et al. 2011).

To avoid reconstruction in the presence of an unhealed infection, repeated aspiration of the joint before the second-stage operation has been recommended (Masterson et al. 1998). However, other studies have shown that repeat aspiration has a limited role in patients who have been treated with parenteral antibiotics (Barrack and Harris 1993, Fehring and Cohen 1996, Koo et al. 2001). We did not perform repeat aspiration before the second-stage operation.

The present study has several limitations. Firstly, although our patients were followed prospectively, the design of the study was a retrospective one—to test our treatment protocol with the hypothesis that it would be successful. Thus, we had no control group with other treatment options. Secondly, because of the small numbers of patients, we could not analyze data for patients stratified according to the virulence of the infecting organism, the type of infection, and the duration of infection. Thirdly, the stem-retaining method may leave a biofilm around the stem, which might mean eventual recurrence of infection.

In summary, we found that second-stage reimplantation after retention of bone-ingrown cementless stems could be an alternative treatment option for infected hip prostheses with well-fixed stems.

## References

[CIT0001] Barrack RL, Harris WH (1993). The value of aspiration of the hip joint before revision total hip arthroplasty. J Bone Joint Surg (Am).

[CIT0002] Beswick AD, Elvers KT, Smith AJ, Gooberman-Hill R, Lovering A, Blom AW (2012). What is the evidence base to guide surgical treatment of infected hip prostheses? systematic review of longitudinal studies in unselected patients. BMC Med.

[CIT0003] Engh CA, Massin P, Suthers KE (1990). Roentgenographic assessment of the biologic fixation of porous-surfaced femoral components. Clin Orthop.

[CIT0004] Estes CS, Beauchamp CP, Clarke HD, Spangehl MJ (2010). A two-stage retention debridement protocol for acute periprosthetic joint infections. Clin Orthop.

[CIT0005] Fehring TK, Cohen B (1996). Aspiration as a guide to sepsis in revision total hip arthroplasty. J Arthroplasty.

[CIT0006] Harris WH (1969). Traumatic arthritis of the hip after dislocation and acetabular fractures: treatment by mold arthroplasty. An end-result study using a new method of result evaluation. J Bone Joint Surg (Am).

[CIT0007] Hsieh PH, Shih CH, Chang YH, Lee MS, Yang WE, Shih HN (2005). Treatment of deep infection of the hip associated with massive bone loss: two-stage revision with an antibiotic-loaded interim cement prosthesis followed by reconstruction with allograft. J Bone Joint Surg (Br).

[CIT0008] Jackson WO, Schmalzried TP (2000). Limited role of direct exchange arthroplasty in the treatment of infected total hip replacements. Clin Orthop.

[CIT0009] Kim YH, Kim VE (1993). Uncemented porous-coated anatomic total hip replacement. Results at six years in a consecutive series. J Bone Joint Surg (Br).

[CIT0010] Koo KH, Yang JW, Cho SH, Song HR, Park HB, Ha YC, Chang JD, Kim SY, Kim YH (2001). Impregnation of vancomycin, gentamicin, and cefotaxime in a cement spacer for two-stage cementless reconstruction in infected total hip arthroplasty. J Arthroplasty.

[CIT0011] Latimer HA, Lachiewicz PF (1996). Porous-coated acetabular components with screw fixation. Five to ten-year results. J Bone Joint Surg (Am).

[CIT0012] Leung F, Richards CJ, Garbuz DS, Masri BA, Duncan CP (2011). Two-stage Total Hip Arthroplasty: How Often Does It Control Methicillin-resistant Infection?. Clin Orthop.

[CIT0013] Lieberman JR, Callaway GH, Salvati EA, Pellicci PM, Brause BD (1994). Treatment of the infected total hip arthroplasty with a two-stage reimplantation protocol. Clin Orthop.

[CIT0014] Lonner JH, Desai P, Dicesare PE, Steiner G, Zuckerman JD (1996). The reliability of analysis of intraoperative frozen sections for identifying active infection during revision hip or knee arthroplasty. J Bone Joint Surg (Am).

[CIT0015] Masri BA, Panagiotopoulos KP, Greidanus NV, Garbuz DS, Duncan CP (2007). Cementless two-stage exchange arthroplasty for infection after total hip arthroplasty. J Arthroplasty.

[CIT0016] Masterson EL, Masri BA, Duncan CP (1998). Treatment of infection at the site of total hip replacement. Instr Course Lect.

[CIT0017] McPherson EJ, Woodson C, Holtom P, Roidis N, Shufelt C, Patzakis M (2002). Periprosthetic total hip infection: outcomes using a staging system. Clin Orthop.

[CIT0018] Tsukayama DT, Estrada R, Gustilo RB (1996). Infection after total hip arthroplasty. A study of the treatment of one hundred and six infections. J Bone Joint Surg (Am).

[CIT0019] Ure KJ, Amstutz HC, Nasser S, Schmalzried TP (1998). Direct-exchange arthroplasty for the treatment of infection after total hip replacement. An average ten-year follow-up. J Bone Joint Surg (Am).

[CIT0020] Wolf CF, Gu NY, Doctor JN, Manner PA, Leopold SS (2011). Comparison of one and two-stage revision of total hip arthroplasty complicated by infection: a markov expected-utility decision analysis. J Bone Joint Surg (Am).

[CIT0021] Yoo JJ, Kwon YS, Koo KH, Yoon KS, Kim YM, Kim HJ (2009). One-stage cementless revision arthroplasty for infected hip replacements. Int Orthop.

